# Immune Regulatory Cell Bias Following Alemtuzumab Treatment in Relapsing-Remitting Multiple Sclerosis

**DOI:** 10.3389/fimmu.2021.706278

**Published:** 2021-10-28

**Authors:** Nicole Kashani, Eve E. Kelland, Borna Vajdi, Lauren M. Anderson, Wendy Gilmore, Brett T. Lund

**Affiliations:** Department of Neurology, Keck School of Medicine, University of Southern California, Los Angeles, CA, United States

**Keywords:** multiple sclerosis, alemtuzumab (Lemtrada), immune regulation, monocyte subsets, regulatory lymphocytes, drug mechanisms

## Abstract

Alemtuzumab is a highly effective treatment for relapsing-remitting multiple sclerosis. It selectively targets the CD52 antigen to induce profound lymphocyte depletion, followed by recovery of T and B cells with regulatory phenotypes. We previously showed that regulatory T cell function is restored with cellular repletion, but little is known about the functional capacity of regulatory B-cells and peripheral blood monocytes during the repletion phase. In this study (ClinicalTrials.gov ID# NCT03647722) we simultaneously analyzed the change in composition and function of both regulatory lymphocyte populations and distinct monocyte subsets in cross-sectional cohorts of MS patients prior to or 6, 12, 18, 24 or 36 months after their first course of alemtuzumab treatment. We found that the absolute number and percentage of cells with a regulatory B cell phenotype were significantly higher after treatment and were positivity correlated with regulatory T cells. In addition, B cells from treated patients secreted higher levels of IL-10 and BDNF, and inhibited the proliferation of autologous CD4^+^CD25^-^ T cell targets. Though there was little change in monocytes populations overall, following the second annual course of treatment, CD14^+^ monocytes had a significantly increased anti-inflammatory bias in cytokine secretion patterns. These results confirmed that the immune system in alemtuzumab-treated patients is altered in favor of a regulatory milieu that involves expansion and increased functionality of multiple regulatory populations including B cells, T cells and monocytes. Here, we showed for the first time that functionally competent regulatory B cells re-appear with similar kinetics to that of regulatory T-cells, whereas the change in anti-inflammatory bias of monocytes does not occur until after the second treatment course. These findings justify future studies of all regulatory cell types following alemtuzumab treatment to reveal further insights into mechanisms of drug action, and to identify key immunological predictors of durable clinical efficacy in alemtuzumab-treated patients.

## Background

Alemtuzumab (Lemtrada, Genzyme, Cambridge, MA) is a humanized monoclonal antibody against CD52, a glycoprotein expressed on the surface of most lymphoid, and to a lesser extent, myeloid cell types. Clinical trials have shown that alemtuzumab is a highly effective treatment for relapsing-remitting multiple sclerosis (RR-MS), improving relapse rate, reducing sustained accumulation of disability and causing long-term remission in a significant number of patients; though this was accompanied by a pronounced risk for development of secondary autoimmunity (1–6).

Alemtuzumab causes rapid and sustained lymphocyte depletion with minimal effects on monocytes, NK cells, dendritic cells and neutrophils (1, 3, 7, 8). Lymphopenia is followed by recovery of different lymphocyte populations with distinct kinetics, though CD4^+^ T cells commonly persist at levels lower than normal limits for a number of years (9–11). The pattern of T-cell repopulation, which is biased toward circulating memory and regulatory T cells (T-regs) and reduced pro-inflammatory cytokine expression, has led many investigators to propose that these are fundamental to the mechanism of action of alemtuzumab (10, 12–16). Indeed, we and others, have observed both an increase in the relative proportion of T-regs following treatment with alemtuzumab, and an increase in the functional capacity of these cells to suppress immune responses (15–20). Even though CD4^+^, CD25^+^, foxP3^+^ T-regs in RR-MS are normal in number and percentage, they exhibit functional deficits (21–23), so restoration of functional competence points to a strong beneficial effect in alemtuzumab treated patients. The effect on T-regs appears to be long term, and is accompanied by significant decreases in numbers and percentages of Th1 and Th17 cells over two years (15–17).

Changes in B cell populations were also reported following treatment with alemtuzumab, as were reductions in innate-like lymphoid cells, plasmacytoid and conventional dendritic cells, and expansion of the immunoregulatory CD56^bright^ NK cell subset (8, 12, 17, 24–26). We have observed, as did Kim and colleagues (25), a significantly increased proportion of B-cells with a regulatory phenotype (B-regs) following treatment with alemtuzumab (17). A large body of literature has emerged in recent years shedding light on the functionality of B-regs and their prevalence not only in healthy populations, but also in disease states (27–30). Critically, evidence of their role in MS has emerged primarily from studies focused on therapeutic interventions (28, 29, 31, 32). However, little is known about the longer-term effect of alemtuzumab treatment on the preponderance of and functionality of B-regs in MS patients. In addition, in spite of clear participation of monocytes and macrophages in the pathogenesis of MS, even less is known about their response to alemtuzumab. Monocytes function as initiators of peripheral inflammatory responses and in the development of central nervous system (CNS) lesions in both MS and its primary animal model, experimental autoimmune encephalomyelitis (33, 34). In non-inflammatory conditions few monocytes can be found within perivascular spaces (34), but this changes in acute inflammatory MS lesions; increased numbers of perivascular and parenchymal monocyte infiltrates are evident together with significant numbers of lymphocytes. In chronic active lesions, monocytes persist as lymphocyte responses diminish (35, 36). In addition, both regulatory and effector functions of blood-derived monocytes have been described in MS (37, 38). Thus, monocytes clearly possess the capacity to both negatively and positively influence the pathology of this disease (39, 40), as well as responses to disease modifying drugs such as alemtuzumab.

In this article, we report data from a study designed to determine whether alemtuzumab treatment significantly alters monocyte phenotype and function, concurrent with altered homeostasis in B-cell and T-cell compartments. The data provide additional insight into the complexity of the response to alemtuzumab revealing that a bias in favor of phenotypic and functional regulatory B and T cells is accompanied by a bias, after second course of treatment, in monocytes producing BDNF and the anti-inflammatory cytokine IL-10. Future studies are needed to understand how these subsets function, both alone and in combination, to control MS disease activity as well as to contribute to the risk for secondary autoimmunity in alemtuzumab-treated patients.

## Methods

### Study Design

This study was a single center, cross-sectional analysis of differences in lymphocyte and monocyte phenotype and function in blood samples collected from distinct cohorts of patients either prior to (0), or 6, 12, 18, 24 or 36 months after their first course of treatment with alemtuzumab. Each cohort was comprised of a unique set of patients with only one blood sample collected at the time of enrollment for each patient. All month 12 samples were collected prior to the second course of treatment with alemtuzumab, and all patients in the 18, 24 and 36 month cohorts had received two courses of treatment with alemtuzumab. This study was reviewed and approved by the University of Southern California (USC), Institutional Review Board prior to commencement, was registered with ClinicalTrials.gov (ID# NCT03647722) and all patients provided written informed consent to participate prior to the collection of blood samples. We enrolled 20 patients that were being evaluated for treatment by their physicians and who qualified to receive alemtuzumab. Blood samples were collected from these patients prior to commencing any therapy. Another 16 patients were enrolled in the 6-month cohort, 20 patients in the 12-month cohort, 20 patients in the 18-month cohort, 20 patients in the 24-month cohort and 16 patients in the 36-month cohort. All were patients at the USC Multiple Sclerosis Comprehensive Care Center, the Los Angeles County/USC Multiple Sclerosis Clinic or the Rancho Los Amigos Medical Center Multiple Sclerosis Clinic. The driving enrollment criteria for this study were prior, or upcoming, treatment with alemtuzumab. There were no longitudinal assessments made in this study, neither immunologically, nor clinically. In addition, this study was carried out independent of patient care and treatment recommendations by clinic physicians; we had no influence on choice of treatment. All patients received the standard treatment course(s) of i.v. alemtuzumab (12 mg/day) for five days at baseline (M0) and three days 12 months later (M12). Thus, the M0 cohort received no treatment, the M6 and M12 cohorts received one treatment, and the M18, M24 and M36 cohorts received both cycles of treatment; no patients in the M24 or M36 cohorts received a third cycle. Enrollment inclusion criteria were as follows: (1) diagnosis of clinically definite relapsing-remitting MS defined by the revised McDonald criteria (Polman et al., 2005, Polman et al., 2010) with an Expanded Disability Status Scale (EDSS) score of 0 to 5.5. (2) qualification for alemtuzumab treatment according to the USC, Department of Neurology, MS Group Clinical alemtuzumab protocol (3) ability to understand and sign the study-specific IRB-approved informed consent form and willingness to donate 80mls of blood for immunological testing on only one occasion. Exclusion criteria were as follows: (1) evidence of progressive disease. (2) clinical diagnosis of other autoimmune disease. (3) patient is of child bearing age with a positive pregnancy test or is unwilling to agree to use a reliable contraceptive method. (4) treatment with any of the following within 30 days of commencing treatment with alemtuzumab or collection of baseline blood sample: Gilenya, Aubagio, Tecfidera. (5) treatment with Natalizumab within 60 days of commencing alemtuzumab treatment or collection of baseline blood sample. (6) treatment with any of the following within 6 months of commencing treatment with alemtuzumab or collection of baseline blood sample: Rituximab, Ocrevus. (7) treatment at any time with any of the following: Mitoxantrone, Cyclophosphamide, Cladribine, Cyclosporine, Azathioprine, Methotrexate or any other immunomodulatory, immunosuppressant or immune homeostasis altering drug. Basic patient cohort characteristics, including age, disease duration, prior medication and time since first course of alemtuzumab treatment, are given in **Table 1**. As this study was designed solely to assess functional immunological measures it was not powered to detect correlations between these immunological and clinical disease measures, as such no clinical assessments were collected in this study.

**Table 1:** Basic characteristics of the different patient cohorts.

Time Cohort(months)	“n”	Age (yrs) at enrollment(mean ± st. dev.)	Months on alemtuzumab(mean ± st. dev.)	Disease duration (yrs.)(mean ± st. dev.)	Medication (# patients) immediately prior to commencing alemtuzumab
0	20	40.03 ± 11.19	0.00 ± 0.00	11.22 ± 7.32	Au (3), C (2), G (5), O (2) P (1), Te (3), Ty (4)
6	16	40.35 ± 11.66	6.42 ± 1.12	11.20 ± 6.94	Au (1), C (2), G (4) Te (3), Ty (6)
12	20	46.78 ± 11.83	12.51 ± 1.16	13.74 ± 6.56	C (2), G (4), R (1) Te (4), Ty (9)
18	20	44.31 ± 10.29	18.80 ± 1.64	12.21 ± 6.57	Au (2), Av (2), G (6), R (1) Te (2), Ty (7)
24	20	47.40 ± 11.08	25.92 ± 1.81	12.26 ± 6.63	Au (2), C (1), G (7), R (1) Te (4), Ty (5)
36	16	50.32 ± 9.07	34.62 ± 2.47	13.11 ± 6.64	Au (3), Av (1), C (1), G (4) R (1), Te (3), Ty (3)

Au, Aubagio; Av, Avonex; C, Copaxone; G, Gilenya; O, Ocrelizumab; P, Plegridy; R, Rituximab; Te, Tecfidera; Ty, Tysabri.

### Blood Collection and FACS Analysis

Approximately 80mls of venous blood were collected in 1x5ml EDTA containing tube, 1x5ml serum tube and 7x10ml sodium heparin containing tubes. As indicated above, blood was collected from patients in the M0 cohort prior to treatment with the first course of alemtuzumab and in the M12 cohort prior to treatment with the second course of alemtuzumab. For all other cohorts the blood was collected within 30-60 days of the desired time point. The absolute numbers of each of the different leukocyte populations were measured from the EDTA sample. The absolute numbers and proportion (percent) of specific subsets of T-cells, B-cells and monocytes were determined using multicolor phenotype panels for fluorescence activated cell sorting (FACS) analyses. We used standard staining procedures, as previously described for whole blood, and intracellular staining for regulatory T-cell assessments (17, 41). The gating strategies used in our laboratories for each of the sub-populations were performed as reported for a similar study (17). All antibodies were purchased from BD Biosciences (San Jose, CA) or Thermofisher (eBiosciences: Waltham, MA). Regulatory B cells were identified as CD19^+^ CD20^+^ CD27^+^ CD24^hi^ CD38^hi^, memory B cells as CD19^+^ CD20^+^ CD27^+^ and total naïve B cells as CD19^+^ CD20^+^ CD27^-^ as previously described (17, 25). Regulatory T-cells were assessed in PBMC isolated from heparinized blood by density gradient centrifugation and identified as CD4^+^ CD25^hi^ CD127^lo/neg^ FoxP3^+^ cells using commercially available kits (eBioscience/ThermoFisher) as described previously (17, 41, 42) and consistent with recommended criteria for Treg identification (43). We classified alternatively-activated non-classical monocytes as CD45^hi^, CD3^-^, CD19^-^, CD56^-^, MHC class II^+^, CD14^low^ CD16^hi^ and conventionally-activated classical monocytes as CD45^hi^, CD3^-^, CD19^-^, CD56^-^, MHC class II^+^, CD14^hi^ CD16^-^. Staining conditions and FACS settings were defined in preliminary experiments that were then used throughout the study. Data acquisition was accomplished using a FACSCanto flow cytometer (BD Biosciences, San Jose, CA) and analyzed using FlowJo software (Ashland, OR). Gating strategies for stained populations were used in combination with appropriate isotype control antibodies for all assays.

### B-Cell Cultures, Monocyte Cultures and Supernatant Collection

B-cells and monocytes were isolated from purified PBMC using commercially available magnetic bead separation protocols according to the manufacturer’s instructions, as previously described (17, 41). CD19-coupled (B-cells) or CD14-coupled (monocytes) magnetic beads (IMag, BD Biosciences, San Diego, CA) were used to positively select B-cells or monocytes, and purity was confirmed as >95% by FACS analyses (data not shown) immediately prior to culture of cells. On the rare occasion when purity was less than 95%, cells were subjected to an additional bead separation step to ensure purity. For generation of culture supernatants, B-cells (10^6^ cells/ml) were stimulated with and without 1µg/ml CD40L (Enzo Life Sciences) and monocytes (10^6^ cells/ml) were stimulated with and without 10µg/ml LPS (Sigma Aldrich, MO) & 100ng/ml IFNγ (BioLegend, San Diego, CA). After 24hrs, supernatants were collected, clarified by centrifugation and stored at -70°C until required for cytokine and growth factor measurements.

### Cytokine and Chemokine Measures

The concentration of solutes was measured in culture supernatant samples using commercially available bead based immunoassays according to manufacturer’s recommendations, as previously reported (41). Flex sets from BD Biosciences (San Diego, CA) were used to measure the concentration of IL-6, IL-10 and IL-12p70, and the Novex™ multiplex assay from ThermoFisher (Grand Island, NY) used to measure the concentration of BDNF. All samples were run in triplicate and the average used to calculate the concentration of each solute based on standard curves of known concentrations. Data were acquired using a BD Accuri C6 flow cytometer (BD Biosciences, San Jose, CA) and analyzed using FlowJo software (Ashland, OR).

### B Cell Functional Assays

T-reg depleted CD4^+^ T cells, used as the targets of the inhibition by B cells, were isolated from purified PBMC using commercially available magnetic bead separation protocols according to the manufacturer’s instructions (Miltenyi Biotec, Auburn, CA), as previously described (17). Briefly, this was accomplished by first isolating CD4^+^ lymphocytes from PBMC using a negative-selection strategy, then positively removing all CD25^+^ cells from the remaining populations using cell-specific magnetic microbeads and magnetic cell separation columns. Success of depletion was confirmed by FACS employing the same strategy as previously reported (17) with up to 95% reduction of CD4^+^ CD25^hi^ cells. T-reg depleted CD4^+^ T cells (2x10^5^ per well) were cultured in triplicate, in wells pre-coated with anti-CD3 (1µg/ml) and anti-CD28 (3µg/ml) (BD Biosciences, San Jose, CA), for 72hrs in the presence or absence of homogeneous populations of autologous B-cells (2x10^5^ per well). Tritiated thymidine (^3^H), 1μCi/well (MP Biomedicals) was added for the last 18 hours and proliferation assessed by measuring incorporation into cellular DNA by liquid scintillation counting. Data were collected using a Wallac MicroBeta TriLux Liquid Scintillation Counter and inhibition expressed as percent reduction in ^3^H-thymidine incorporation in T-reg depleted CD4^+^ T cells co-cultured with autologous B-cells as compared to culture alone.

### Statistical Analyses

Statistical analyses were carried out using SigmaStat software, version 4.0 (Systat Software Inc., San Jose, CA). Differences between the pre-treated and treated cohorts were compared for significance using a *t-test* for normally distributed data, or Mann-Whitney Rank Sum Test if the assumptions for parametric procedures were not satisfied (significance of differences between the pre-treated cohort and each treated cohorts is noted above a solid black line in each graph). Differences over time were assessed by One Way Analysis of Variance for normally distributed data, or Kruskal-Wallis One Way Analysis of Variance on Ranks for non-parametric data. Correlation between measures was assessed using the Spearman’s Rank-Order correlation, with accompanying p-value and rho (r) added to the plot of each associated measures. P-values were calculated on raw data with values of 0.05 or less considered significant and depicted in the figures as * = p<0.05; ** = p<0.001 and *** = p<0.0001 versus the pre-treated cohort value.

## Results

### Measures of B Cell Phenotypes

**Figure 1:**
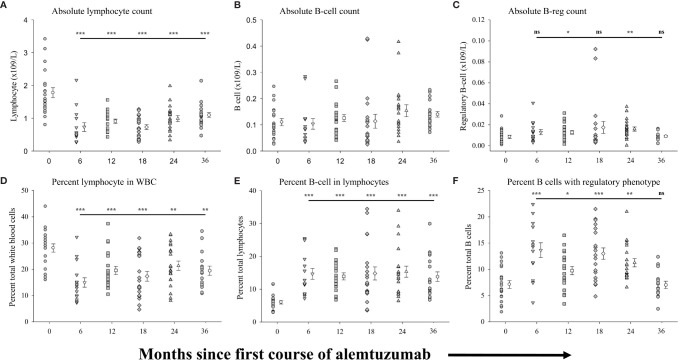
Measures of peripheral blood regulatory B-cells following alemtuzumab treatment: Panels **(A–F)** illustrate different measures for each treatment cohort (6 month = inverted triangle; 12 month = square; 18 month = diamond; 24 month = triangle; 36 month = hexagon) as compared with a pre-treated patient cohort (circles). Panel **(A)** shows the absolute number of lymphocytes per liter of blood. Panel **(B)** shows the absolute number of CD19^+^ B lymphocytes per liter of blood. Panel **(C)** shows the absolute number of CD19^+^ CD20^+^ CD27^+^ CD24^hi^ CD38^hi^ regulatory B lymphocytes per liter of blood. Panel **(D)** shows the percent of lymphocytes found in total white blood cells. Panel **(E)** shows the percent of CD19^+^ B lymphocytes found within the total lymphocyte population. Panel **(F)** shows the percent of CD19^+^ with a CD20^+^ CD27^+^ CD24^hi^ CD38^hi^ regulatory phenotype. Data for each cohort in each panel shows the individual values for each patient in the cohort (filled circles, filled triangles etc.) adjacent to the mean ( ± SEM) for the group as a whole (open circles, open triangles etc.). Significant changes compared to the pre-treated patient cohort are identified as *** (p≤ 0.001), ** (p ≤ 0.01), * (p ≤ 0.05) or ns (not significant), and are plotted above the corresponding cohort on top of the solid back line.

**Figure 1** shows the differences in absolute numbers and proportions (percent of parent population) of total lymphocytes (**Figures 1A, D**), CD19^+^ B-cells (**Figures 1B, E**) and regulatory B-cells (**Figures 1C, F**) found in the peripheral blood of patients prior to, and at the various time points following, treatment with alemtuzumab (see **Supplementary Table 1** for data values). In agreement with reported data we observed a significantly lower (p<0.001) average number of circulating lymphocytes (**Figure 1A** and **Supplementary Table 1**) in each cohort of patients after alemtuzumab treatment compared with patients in the pre-treated cohort. In contrast to the absolute numbers of lymphocytes, we did not observe significant differences in the absolute numbers of B cells (**Figure 1B** and **Supplementary Table 1**) in any of the cohorts. Though there was a trend for increasing numbers of B cells in the 24- and 36-month cohorts this did not reach statistical significance. The absolute numbers of regulatory B cells (**Figure 1C** and **Supplementary Table 1**) however trended higher in all of the treatment cohorts as compared to pre-treated patients reaching statistical significance in the 12-month (p=0.047) and 24-month cohorts (p=0.005). Differences in the proportion of lymphocytes (**Figure 1D**) and B cells (**Figure 1E**) within parent populations were similar to published reports (9–11). The percentage of lymphocytes within total white blood cells (**Figure 1D** and **Supplementary Table 1**) was significantly lower (p<0.001) in the 6-month cohort, almost half the size of the pre-treated cohort and remained similarly significantly lower in each of the other cohorts. In contrast, the percent of lymphocytes with a B cell phenotype (**Figure 1E** and **Supplementary Table 1**) was significantly higher (p<0.001) in all treated cohorts compared with pre-treated patients, more than doubling in size. Within the B cell compartment there was a bi-phasic increase in the proportion of regulatory B cells (**Figure 1F** and **Supplementary Table 1**), which showed similar trends to our published observations with cryopreserved cells (17). We saw a significantly higher proportion of regulatory B cells in the 6-month (p<0.001), 12-month (p=0.018), 18-month (p<0.001) and 24-month (p<0.001) cohorts. However, in the 36-month cohort, the percent of B cells with a regulatory phenotype was reduced, matching that of the pre-treated cohort.

### Measures of Regulatory B-Cell Function

**Figure 2:**
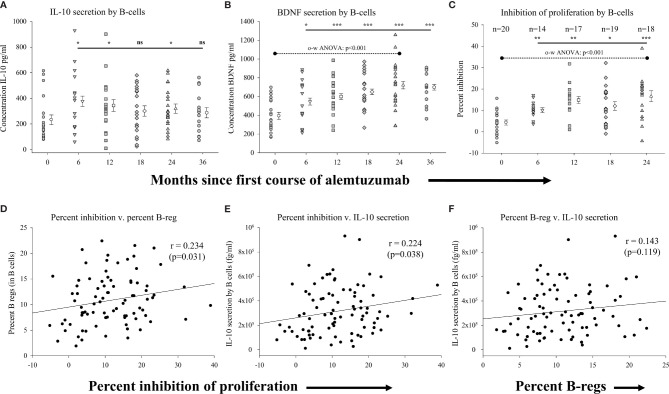
Measures of regulatory B-cell function following alemtuzumab treatment: Panels **(A–C)** illustrate different measures for each treatment cohort (6 month = inverted triangle; 12 month = square; 18 month = diamond; 24 month = triangle; 36 month = hexagon) as compared with a pre-treated patient cohort (circles). Panels **(A)** (IL-10) & **(B)** (BDNF) show the concentration (pg/ml) of solutes secreted by stimulated CD19^+^ B lymphocytes. Panel **(C)** shows the percent of inhibition of T cell proliferation by purified CD19^+^ B lymphocytes. Data for each cohort in panels **(A–C)** shows the individual values for each patient in the cohort (filled circles, filled triangles etc.) adjacent to the mean ( ± SEM) for the group as a whole (open circles, open triangles etc.). Significant changes compared to the pre-treated patient cohort are identified as *** (p≤ 0.001), ** (p ≤ 0.01), * (p ≤ 0.05) or ns (not significant), and are plotted above the corresponding cohort on top of the solid back line. One-way ANOVA (panel B) with corresponding p-value is shown with a dashed line across the top of the data points tested. Panels **(D–F)** show scatter plots and Spearman’s Rank-Order correlations (rho (r)) of the **(D)** percent inhibition of T cell proliferation (x-axis) versus percent of regulatory B lymphocytes, **(E)** percent inhibition of T cell proliferation (x-axis) versus concentration of IL-10 and **(F)** percent of regulatory B lymphocytes versus concentration of IL-10 for all data points in the study.

We assessed the function of B cells isolated from the different cohorts, including the relative capacity of B cells to secrete anti-inflammatory solutes (IL-10), their capacity to secrete stimulants of neural growth (BDNF) and their capacity to inhibit T cell proliferation (see **Supplementary Table 2** for data values). We assessed the secretion of solutes from homogeneous populations of CD19^+^ B cells (>95-98% purity) cultured in the presence or absence of CD40-ligand. **Figure 2A** (**Supplementary Table 2**) shows that significantly higher levels of IL-10 were secreted by B cells from patients in the 6-month (p=0.024), 12-month (p=0.044) and 24-month cohorts p=0.041) as compared to the pre-treated cohort. Though still trending higher, IL-10 levels secreted by B cells from patients in the 18-month and 36-month cohorts were not statistically significantly different than the pre-treated cohort. The concentration of BDNF (**Figure 2B**; **Supplementary Table 2**) secreted by B cells was also significantly higher in each of the treatment cohorts as compared to the pre-treated cohort with BDNF secretion by CD19^+^ B cells increasing significantly (p<0.001; one-way ANOVA) with time on alemtuzumab through the 24-month cohort. We also assessed the capacity of B cells to inhibit the proliferation of target cells (autologous CD4^+^, CD25^-^ T cells) in all samples where sufficient B cells could be harvested. **Figure 2C** (**Supplementary Table 2**) shows that there was significantly greater inhibition of proliferation in all treated cohorts up to 24 months, as compared to the pre-treated cohort. There was on average only a 4.33 ± 1.18% reduction in the proliferation of purified CD4^+^, CD25^-^ isolated from patients in the pre-treated cohort when co-cultured with an equal number of autologous B cells isolated from the same blood sample. In contrast, there was a significantly (p=0.002) higher, ~2.5-fold, inhibition of proliferation in the 6-month cohort (10.09 ± 1.11%). The percent of inhibition was also significantly higher in the 12-month (p<0.001), 18-month (p=0.014) and 24-month cohorts (p<0.001) as compared to the pre-treated cohort. The greater levels of inhibition of proliferation increased with time on alemtuzumab and were highly significant (p<0.001; one-way ANOVA).

We showed that there was not only an increased capacity of B-cells to secrete the anti-inflammatory cytokine IL-10 following treatment with alemtuzumab, but that these cells were also functionally able to inhibit the proliferation of CD4^+^, CD25^-^ T cells. We therefore assessed if there was a correlation in the capacity to inhibit proliferation by B cells with firstly the percent of regulatory B cells within this population, and secondly with the capacity of B cells to secrete IL-10. For these analyses we plotted the data obtained in all assays regardless of the study cohort. These data show that was there was a significant, positive, albeit weak, association (rho=0.234; p=0.031) between the capacity of B-cells to inhibit the proliferation and the percent of B-cells with a regulatory phenotype (**Figure 2D**). Not surprisingly, there was also a similarly significant, positive, association (rho=0.224; p=0.038) between the levels of IL-10 secreted by B-cells and the capacity of B-cells to inhibit the proliferation of T-cells (**Figure 2E**). There was also a weakly positive but not statistically significant association (rho=0.143; p=0.119) between the percent of B-cells with a regulatory phenotype and the levels of IL-10 secreted by B-cells (**Figure 2F**). In contrast to the correlative assessments with measures of percent of B-cells, there was no correlation observed with measure of absolute numbers of regulatory B cells (data not shown).

### Measures of Peripheral Blood Regulatory T-Cells

**Figure 3:**
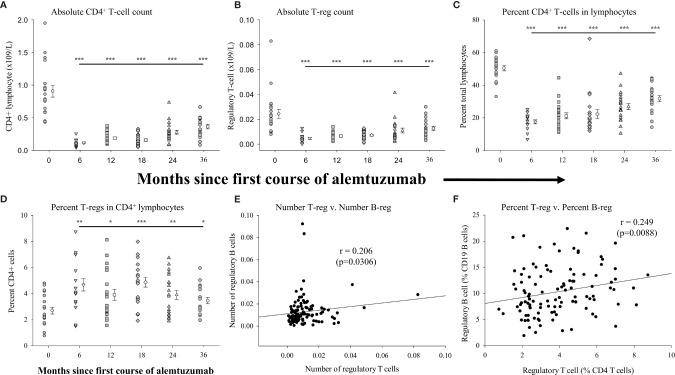
Measures of peripheral blood regulatory T-cells following alemtuzumab treatment: Panels **(A–D)** illustrate different measures for each treatment cohort (6 month = inverted triangle; 12 month = square; 18 month = diamond; 24 month = triangle; 36 month = hexagon) as compared with a pre-treated patient cohort (circles). Panel **(A)** shows the absolute number of CD4^+^ T lymphocytes per liter of blood. Panel **(B)** shows the absolute number of CD4^+^ CD25^hi^ CD127^lo/neg^ FoxP3^+^ regulatory T lymphocytes per liter of blood. Panel **(C)** shows the percent of CD4^+^ T lymphocytes found within the total lymphocyte population. Panel **(D)** shows the percent of CD4^+^ with a CD25^hi^ CD127^lo/neg^ FoxP3^+^ regulatory phenotype. Data for each cohort in panels **(A–D)** shows the individual values for each patient in the cohort (filled circles, filled triangles etc.) adjacent to the mean ( ± SEM) for the group as a whole (open circles, open triangles etc.). Significant changes compared to the pre-treated patient cohort are identified as *** (p≤ 0.001), ** (p ≤ 0.01), * (p ≤ 0.05) or ns (not significant), and are plotted above the corresponding cohort on top of the solid back line. Panels **(E, F)** show scatter plots and Spearman’s Rank-Order correlations (rho (r)) of the number of regulatory T lymphocytes (x-axis) versus the number of regulatory B lymphocytes **(E)** or percent of regulatory T lymphocytes (x-axis) versus the percent of regulatory B lymphocytes **(F)** for all data points in the study.

In an effort to more comprehensively evaluate immunological biases following treatment with alemtuzumab we included assessments of differences in regulatory T cells in our study. **Figure 3** (see **Supplementary Table 3** for data values) shows the measures of regulatory T-cells (CD45^hi^, CD3^+^, CD4^+^, CD25^int/hi^, CD127^lo/neg^ and FoxP3^+^) in the same samples of peripheral blood in which B cells were assessed. We have already shown that there was a significant reduction in the absolute numbers (**Figure 1A**) and percent of total lymphocytes (**Figure 1D**) in all of the treated patient cohorts. Similar to published reports, there was also a significant reduction (p<0.001 in all cohorts) in the absolute numbers (**Figure 3A** and **Supplementary Table 3**), and proportion (percent) of CD4^+^ lymphocytes (**Figure 3C** and **Supplementary Table 3**) in all of the treated patient cohorts. We also assessed differences in the absolute numbers of regulatory T cells in the multiple cohorts. **Figure 3B** (**Supplementary Table 3**) shows that, in comparison to the number of regulatory T cells in the pre-treated cohort there was a significantly reduced number of regulatory T cells in each of the 6-month (p<0.001), 12-month (p<0.001), 18-month (p<0.001), 24-month (p<0.001) and 36-month cohorts (p=0.008). In contrast to the reduction in the numbers of regulatory T cells there were significantly larger proportions of regulatory T cells within the CD4^+^ lymphocyte population in the alemtuzumab-treated cohorts (**Figure 3D** and **Supplementary Table 3**). In patients from the 6-month cohort, 4.67 ± 0.47% of CD4^+^ lymphocytes were phenotypically regulatory T cells as compared to the pre-treated cohort where there were only 2.69 ± 0.26%, a significant (p=0.007) and almost 2-fold increase. The percent of regulatory T cells remained significantly higher than the pre-treated cohort in the 12-month (p=0.048), 18-month (p<0.001), 24-month (p=0.008) and 36-month cohorts (p=0.038).

Given the similar patterns observed in the percent of lymphocytes that were regulatory B cells and regulatory T cells we assessed if there was any correlation between these measures. For this analysis we plotted the data obtained from all samples, regardless of the study cohort. These data show that there was a significant positive association (rho=0.206: p=0.0306) between the numbers of regulatory T cells and the numbers of regulatory B cells in all of the samples collected (**Figure 3E**). There was an even stronger association (rho=0.249: p=0.0088) between the percent of regulatory T cells and the percent of regulatory B cells with the parent population (CD4^+^ lymphocytes and CD19^+^ lymphocytes respectively) (**Figure 3F**).

### Measures of Peripheral Blood Monocytes

**Figure 4:**
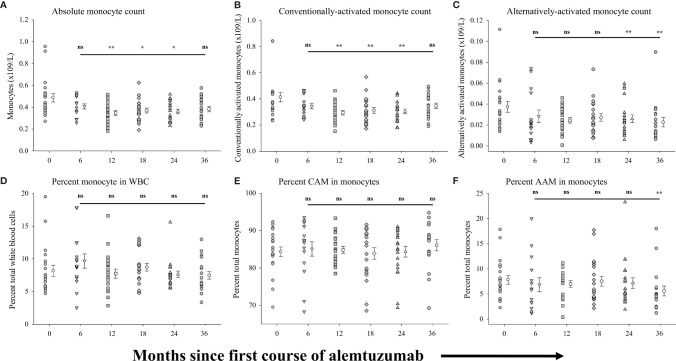
Measures of peripheral blood monocytes following alemtuzumab treatment: Panels **(A–F)** illustrate different measures for each treatment cohort (6 month = inverted triangle; 12 month = square; 18 month = diamond; 24 month = triangle; 36 month = hexagon) as compared with a pre-treated patient cohort (circles). Panel **(A)** shows the absolute number of CD14^+^ monocytes per liter of blood. Panel **(B)** shows the absolute number of conventionally-activated monocytes (CAM; CD14^hi^ CD16^-^) per liter of blood. Panel **(C)** shows the absolute number of alternatively-activated, non-classical monocytes (AAM; CD14^low^ CD16^hi^) per liter of blood. Panel **(D)** shows the percent of CD14^+^ monocytes found in total white blood cells. Panel **(E)** shows the percent of conventionally-activated monocytes (CD14^hi^ CD16^-^) found within the total monocyte population. Panel **(F)** shows the percent of alternatively-activated, non-classical monocytes (CD14^low^ CD16^hi^) found within the total monocyte population. Data for each cohort in each panel shows the individual values for each patient in the cohort (filled circles, filled triangles etc.) adjacent to the mean ( ± SEM) for the group as a whole (open circles, open triangles etc.). Significant changes compared to the pre-treated patient cohort are identified as ** (p ≤ 0.01), * (p ≤ 0.05) or ns (not significant), and are plotted above the corresponding cohort on top of the solid back line.

We analyzed total peripheral blood monocytes and subsets of conventionally-activated monocytes and alternatively-activated monocytes. **Figure 4** shows the measures of these populations in the same samples of peripheral blood from which the above B cell and T cell data were generated. In all of the alemtuzumab-treated patient cohorts the numbers of circulating monocytes (**Figure 4A**, see **Supplementary Table 4**) were somewhat reduced compared to the pre-treated cohort. Patients from the 12-month (p=0.004), 18-month (p=0.014) and 24-month cohorts (p=0.008) had significantly fewer monocytes compared to the pre-treated cohort, and whereas still lower in both the 6-month cohort and 36-month cohort this was not significantly different. Similar trends were observed across the different cohorts in the absolute numbers of conventionally-activated monocytes (CAM: **Figure 4B**) and alternatively-activated phenotype (AAM: **Figure 4C**). In contrast to the minor differences between some cohorts in the numbers of monocytes, the proportion (percent) of monocytes found within the total white blood cell population was not significantly different in any of the treated cohorts as compared to the pre-treated cohort (**Figure 4D**). There were similarly no significant differences between any cohorts in the percent of monocytes that were CAM (**Figure 4E**), though there were wide variations in the values observed. We also detected no significant differences in the percent of monocytes that were AAM (**Figure 4F**) between the pre-treated cohort and each of the 6-, 12-, 18- and 24-month cohorts. There was however a significant (p=0.025) reduction in the percent AAM in the 36-month cohort (5.60 ± 0.99%) as compared to the pre-treated cohort (7.83 ± 0.89%).

**Figure 5:**
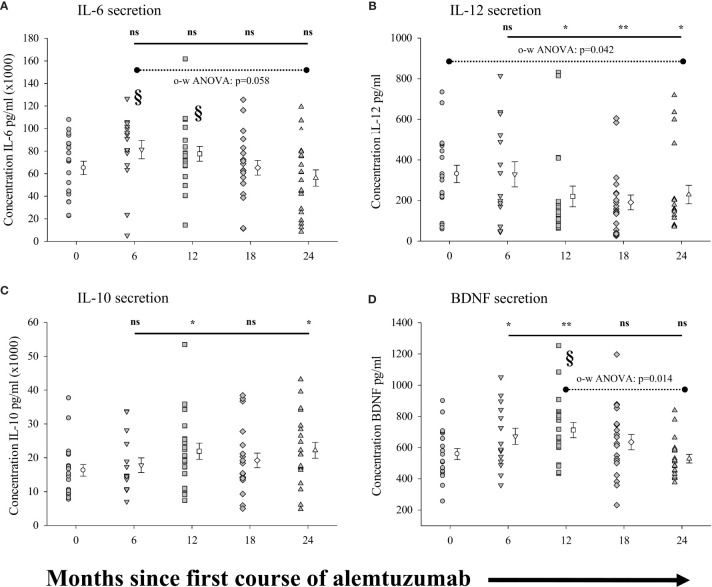
Measures of peripheral blood monocyte function following alemtuzumab treatment: Panels **(A–D)** illustrate different measures for each treatment cohort (6 month = inverted triangle; 12 month = square; 18 month = diamond; 24 month = triangle; 36 month = hexagon) as compared with a pre-treated patient cohort (circles). Panels **(A)** (IL-6), **(B)** (IL-12), **(C)** (IL-10) & **(D)** (BDNF) show the concentration (pg/ml) of solutes secreted by stimulated CD14^+^ B monocytes. Data for each cohort in each panel shows the individual values for each patient in the cohort (filled circles, triangles etc.) adjacent to the mean ( ± SEM) for the group as a whole (open circles, triangles etc.). Significant changes compared to the pre-treated patient cohort are identified as ** (p ≤ 0.01), * (p ≤ 0.05) or ns (not significant), and are plotted above the corresponding cohort on top of the solid back line. Significant differences compared to the 24-month cohort are identified as § (p<0.05). One-way ANOVA **(A, B, D)** with corresponding p-value is shown with a dashed line across the top of the data points tested.

Because the phenotype of a given cell population does not necessarily equate to the function of these cells, we also measured the capacity of purified (>95%) populations of CD14^+^ monocytes to secrete the anti-inflammatory cytokine IL-10, the pro-inflammatory cytokines IL-6 and IL-12p70, and the neural growth factor BDNF in response to stimulation. The results are shown in **Figure 5** (see **Supplementary Table 4** for all data values). No significant differences were observed in the concentration of IL-6 secreted by monocytes (**Figure 5A**) from patients in any of the treatment cohorts as compared to the pre-treated cohort. There was however a significantly lower concentration of IL-6 (p=0.014: §) secreted by monocytes from patients in the 24-month cohort as compared to the 6-month cohort. Interestingly, there was also a trend for the secretion of lower concentrations of IL-6 in each of the patient cohorts with increased time on alemtuzumab (12, 18 and 24 month), though this did not reach significance (p=0.058, one-way ANOVA). In contrast to IL-6, IL-12p70 secretion by monocytes (**Figure 5B**, see **Supplementary Table 4**) was significantly reduced in patient cohorts with increased time since the first course of alemtuzumab (p=0.042, one-way ANOVA). Though there was no significant reduction in the concentration of IL-12p70 secreted by monocytes from patients in the 6-month cohort, IL-12p70 secretion by monocytes was significantly reduced in patients from the 12-month (p=0.032), 18-month (p=0.009) and 24- month cohorts (p=0.047). For the anti-inflammatory cytokine IL-10 (**Figure 5C**, see **Supplementary Table 4**), we observed a biphasic increase in the levels of IL-10 secretion by monocytes in the 12-month and 24-month treated cohorts. Though elevated, the levels of IL-10 from the 6-month and 18-month cohorts were not significantly elevated as compared to the pre-treated cohort. However, significantly more IL-10 was secreted by monocytes from the 12-month (p=0.041) and 24-month cohorts (p=0.025). We also observed significantly higher secretion of the neural growth factor BDNF by stimulated monocytes (**Figure 5D**, see **Supplementary Table 4**) in those cohorts who had received only the first course of alemtuzumab (6 month, p=0.042 & 12 month, p=0.015) as compared to the pre-treated cohort. In contrast, BDNF secretion in those cohorts who had received a second course of alemtuzumab (18- and 24-month) was not significantly different as compared to the pre-treated cohort. Interestingly, a one-way ANOVA demonstrated the significance of the differences in secretion of BDNF across the 12-, 18- and 24-month cohorts (p=0.014), with the level of secretion of BDNF in the 24- month cohort significantly lower than that observed in the 12 month cohort (p=0.011: §).

We lastly ran a series of analyses of the measures of each of the monocyte populations (all monocytes, CAM and AAM) including secreted solutes to see if they correlated with all measures of the regulatory T cell and regulatory B cell populations in the same samples. Not surprisingly, given the different patterns in the various cohorts, we observed no correlations between any of the measures of myeloid and lymphoid sub-populations (data not shown).

## Discussion

Here we present data that critically adds to our published findings (17) describing the effect of alemtuzumab treatment of the phenotype and function of regulatory lymphocyte subsets in multiple sclerosis patients. Our results confirm increased representation of regulatory B cells within the B cell compartment (**Figure 1F**) in patients treated with alemtuzumab through at least 2 years following the first treatment cycle. In addition, we show for the first time that B cells from multiple sclerosis patients treated with alemtuzumab have increased anti-proliferative function (**Figure 2C**), that this correlates with IL-10 secretion (**Figure 2E**), and that increases in regulatory B cells correlate with increases in regulatory T cells (**Figures 3E, F**). Furthermore, we also describe changes in the functional capacity of peripheral blood monocytes that can also be detected following treatment with alemtuzumab (**Figure 5**). Indeed, while there was little change in the number and proportion of monocytes (**Figure 4**), there was a discernable anti-inflammatory bias in the monocyte response to stimulation, but only in patients who have received both of the annual cycles of alemtuzumab treatment.

It is important to note that this study was designed to assess functional immunological measures across unique cohorts of alemtuzumab-treated patients in a cross-sectional, and not longitudinal design. As such, it was not powered to detect correlations between immunological and clinical disease measures. This design has obvious limitations; however, even in the largest study published to date, in which immunological changes were assessed longitudinally in 802 patients over a two year period following alemtuzumab treatment, immunological correlations with measures of clinical efficacy were not detected (44). Thus the capacity to detect an immune efficacy signature remains elusive and may necessitate a unique approach in future analyses.

We previously showed an increased proportion of regulatory B cells within the B cell compartment of cryopreserved PBMC collected from patients at various time points following alemtuzumab treatment (17), consistent with published observations (12, 24, 45). In this report we not only confirmed these observations, but also showed enhanced functional recovery of B cells both in measures of secreted solutes and in the capacity to inhibit proliferative responses. Though regulatory B cell increases have previously been reported following treatment with alemtuzumab (24, 25) we show, for the first time, that this was also accompanied by an increase in the functional capacity of these cells. This was robust and observed in each cohort of patient tested through the 24-month cohort. With increased time following the first course of treatment with alemtuzumab, B cells showed an increasing capacity to inhibit the proliferation of autologous CD4^+^ T cells, with a greater than 4-fold increase in inhibition observed in the 24-month cohort. The assay used in these assessments has, to our knowledge, not been reported in the literature. We made use of total B cells, which, in the absence of purification into B cell subsets, did not allow precise identification of the inhibitory B cell subset. Unfortunately, this assay strategy was necessary because of limitations in the volume of blood collected, the use of blood for multiple assays, and the fact that patients exhibited significant leukopenia. This limitation could be addressed in future studies that focus on the B cell compartment by isolation of regulatory cells to homogeneity for use in such an assay. Nevertheless, the assays were conducted in the exact same manner using identical culture conditions across all experiments, lending validity and credibility to differences in measures between the cohorts. Although the mechanism of proliferation inhibition, be it *via* cell-cell contact, secretion of regulatory solutes, or a combination of both, was not investigated in this study, we did note that the level of inhibition of proliferation by B-cells correlated not only with the percent of regulatory B cells, but also with the propensity of B cells to secrete IL-10 when stimulated. Indeed, there was a trend toward increased IL-10 secretion by B-cells in all treatment cohorts the role of which could be confirmed in future studies by inclusion of an IL-10 neutralizing antibody in control proliferation assays.

Interestingly, there was also an increase in secretion of the neurotrophic factor BDNF by stimulated B-cells through the 24-month cohort, though this appeared to taper off in the 36-month cohort. Similarly, the absolute number and percent of regulatory B cells in the 36-month cohort were less than other treatment cohorts and more closely approximated that of the pre-treated cohort. This may suggest that regulatory B cells play a role in the mechanism of action of alemtuzumab in the earlier stages of re-emergence of this population, following each course of treatment. It would be interesting to assess the functionality of these cells in patients who had received a 3^rd^ or even 4^th^ course of treatment with alemtuzumab. Furthermore, the enhanced BDNF response in the earlier treatment cohorts may reflect a durable change in function of B-cells, which could have implications for clinical efficacy and may allow the use of BDNF secretion as a surrogate marker.

Though CD19^+^ B cells are rapidly depleted following alemtuzumab treatment they are the first cells to replete fully and, on average, by 6-12 months are found at higher levels than baseline (1, 3, 12, 24, 26). However, we (17), and others (24–26), have shown that recovered B-cells are essentially devoid of memory cells, and are composed mostly of immature and mature naïve B-cells. It has been suggested that these B-cell dynamics are central to the risk for secondary autoimmunity, and that this rapid repopulation in the absence of effective T-cell regulation is a key factor in the development of autoimmunity (12). Notably absent in this discussion is the fact that regulatory B-cells are found within the CD19^+^, CD27^-^ naïve B-cell population that predominate following alemtuzumab treatment (32, 46), and that regulatory B-cells are purported to modulate disease severity (47). Our data presented here clearly show that these cells are increasingly functional following alemtuzumab treatment and add another twist to the ever evolving role of these cells in disease pathology, quiescence and even treatment side-effects.

Our assessment of T cell function focused solely on regulatory T cells. Prior work from our laboratories, and that of others, has described significant reductions in numbers and percentages of effector T cells and reduced effector to regulatory T cell ratios (9, 15, 17, 42). The changes in regulatory T cells measures observed here were comparable to our prior observations (17) and other published data showing increased representation of these cells for up to 2 years following alemtuzumab treatment as compared to the pre-treated cohort (9, 15, 16, 42). We and others have previously shown a rebound in regulatory T cell function (15, 17, 42), and though it would have been preferable, for the overall outcome from this study, to replicate the rebound proliferation of CD4^+^, CD25^-^ T cells as published in our prior work, we were again limited by cell recovery. Our data however, clearly show that the absolute numbers of circulating regulatory T cells drops in alemtuzumab treated patients, in contrast to the absolute numbers of regulatory B cells. It should be noted however, that although there was an 8-fold drop in the number of CD4^+^ lymphocytes in the 6-month cohort as compared to baseline, there was a less than 5-fold drop in the number of regulatory T cells in the same patients. This translates into an almost 2-fold increase in the percent of regulatory cells within the CD4^+^ lymphocyte compartment. Regulatory T cells and regulatory B-cells showed similar kinetics of change, with a biphasic increase occurring soon after each of the two courses of treatment with alemtuzumab, and measures of both absolute number and percent of parental population showed significant correlations. The multi-faceted regulatory immune network that cross-activates and cross-regulates has been well described (48–57) and it would be interesting to know if these two regulatory populations are functionally inter-dependent at the different stages of repletion. Do they stimulate each other, or are they independently increased as a more general default regulatory program in the response to lymphopenia?

Once characterized as a programmed, stereotypic response to injury, monocyte cell activation is now recognized as a more complex series of responses to distinct stimuli within the microenvironment. The belief that activated myeloid cells simply exacerbate inflammatory responses has been successfully challenged, with monocytes and macrophages now categorized as either classically activated or alternatively activated cells (39, 40). Whereas classical activation of cells, observed in a pro-inflammatory state, is characterized by increased expression of solutes such as TNF, IL-6 and IL-1β, alternatively activated cells secrete anti-inflammatory cytokines such as IL-4, IL-10 and TGFβ, and express factors involved in tissue repair and reconstruction (39, 40). Our assessments of monocytes showed there was little change in the overall numbers of total monocytes or sub-populations of classically activated monocytes and alternatively activated monocytes. While we did observe significant reduction in the absolute numbers of circulating monocytes in the 12-month, 18-month and 24-month cohorts, these differences were minor, and not outside the variability seen in treated populations at similar time points following treatment (8, 10, 45). The use of cross-sectional data, with the wider than anticipated variance in the values for pre-treated patients, is most likely responsible for these differences as compared to published data. In contrast to the change in absolute numbers, there were no significant differences in any of the cohorts in the percent of monocytes, or in the relative composition of the monocyte population. However, in assessing the capacity to secrete various solutes we clearly observed functional differences in the later treatment cohorts, not only compared to the pre-treated patients, but also when compared to earlier treatment cohorts. Overall, in the cohort of patients assessed 6 months after receiving the first course of alemtuzumab treatment, monocytes were somewhat static in their response to stimuli, with little difference in secretion of pro-inflammatory and anti-inflammatory solutes. However, in those cohorts analyzed after the second course of treatment with alemtuzumab there was a general increase in the level of secretion of IL-10 accompanied by reduced levels of secretion of pro-inflammatory cytokines IL-12 & IL-6. This change to an anti-inflammatory bias in the secretory profile of monocytes clearly reflects a different kinetic response to treatment with alemtuzumab in monocytes as compared to lymphocytes, in which regulatory populations and functions increase within the first 6 months of treatment. BDNF secretion by monocytes was increased following the first cycle of alemtuzumab, and while we cannot say what if there is a direct or specific effect of this monocyte-derived BDNF our interpretation is that this contributes to a regulatory environment that favors regulatory subsets in lymphocytes. Additional studies are required to confirm a direct interaction among monocytes, T cells and B cells in which increased functional capacity requires or is dependent upon BDNF. Interestingly, lymphocytes from alemtuzumab treated patients also produce higher levels of BDNF and CNTF in response to myelin basic protein stimulation *in vitro* (58).

As far as we are aware this is the first time that the function of monocytes has been assessed in patients following treatment with alemtuzumab; it is intriguing therefore to speculate as to the utility of these measures. A large proportion of patients treated with alemtuzumab develop secondary autoimmunity, which occurs later in the treatment course, peaking 2 to 3 years after the first treatment (1–6). Though not typically seen with other MS therapies, secondary autoimmune diseases have been shown to be a consequence of lymphopenia and lymphocyte depletion in a variety of clinical applications or diseases, including HIV infection, transplantation strategies and treatment for various malignancies (59, 60). In this respect, it is somewhat surprising that disease activity in MS, believed to be driven in part by autoimmune activity, remains suppressed while secondary autoimmune diseases are activated, or, show a lower threshold for activation. One explanation may be that the action of alemtuzumab selectively targets active ongoing autoimmune mechanisms rather than general immune activities for prolonged suppression.

In our recent study, and that of Wiendl and colleagues, there were no obvious immunological metrics that could predict secondary autoimmunity (17, 44). Though we did not assess any clinical metrics in this cross-sectional study, and therefore cannot assess any direct correlations, it is interesting, given this new data, to speculate a role for myeloid cells. Though monocytes have been enumerated in alemtuzumab studies, an association between monocyte function and secondary autoimmunity has not been addressed. Our data showed that the most significant changes in immunological bias in monocytes were seen after the second course of treatment: the later cohorts (18-month and 24-month) as a whole showed significantly reduced IL-12p70 secretion by monocytes, accompanied by increased IL-10 secretion. This however was not a one size fits all response, and there were clear outliers in these cohort secreting high levels of IL-12p70 and low IL-10. Perhaps a lack of alemtuzumab-associated changes in peripheral-blood monocyte function to an anti-inflammatory immunological bias might pre-dispose patients to developing secondary autoimmunity, and may be related to their roles as antigen presenting cells for both T and B-cell responses (61–63).

## Data Availability Statement

The raw data supporting the conclusions of this article will be made available by the authors upon reasonable request.

## Ethics Statement

The studies involving human participants were reviewed and approved by the University of Southern California Health Sciences Campus Institutional Review Board, Los Angeles, CA. The patients/participants provided their written informed consent to participate in this study.

## Author Contributions

BL, EK, and WG were involved in the development of the study concept and design, analysis and interpretation of data and in the drafting and revision of the manuscript. NK, BV, LA, EK, and BL all had a major role in the recruitment of patients to the study and in the acquisition of data. All authors contributed to the article and approved the submitted version.

## Funding

This study was supported by Sanofi/Genzyme Corporation (GZ-2017-11746) to BL. Sanofi Genzyme had no role in the design of the study nor in the analyses or interpretation of the data.

## Conflict of Interest

BL has received funding from Novartis Pharmaceuticals Corporation and Teva Pharmaceuticals Corporation for investigator-initiated research unrelated to this work and has received honoraria from Teva Pharmaceuticals Corporation unrelated to this work. EK has received funding for investigator-initiated research from Novartis Pharmaceuticals Corporation and Teva Pharmaceuticals Corporation investigator-initiated research unrelated to this work. WG has received funding from Sanofi Genzyme for investigator-initiated research unrelated to this work and honoraria for service on advisory and scientific boards for Sanofi Genzyme.

The remaining authors declare that the research was conducted in the absence of any commercial or financial relationships that could be construed as a potential conflict of interest.

## Publisher’s Note

All claims expressed in this article are solely those of the authors and do not necessarily represent those of their affiliated organizations, or those of the publisher, the editors and the reviewers. Any product that may be evaluated in this article, or claim that may be made by its manufacturer, is not guaranteed or endorsed by the publisher.
